# How Much Are People Willing to Pay for Clean Air? Analyzing Housing Prices in Response to the Smog Free Tower in Xi’an

**DOI:** 10.3390/ijerph181910210

**Published:** 2021-09-28

**Authors:** Haiyong Zhang, Sanqin Mao, Xinyu Wang

**Affiliations:** 1 School of Mathematics and Finance, Chuzhou University, Chuzhou 239000, China; haiyongzhang@chzu.edu.cn; 2 School of International and Public Affairs, Shanghai Jiao Tong University, Shanghai 200030, China; 3 School of Economics and Management, China University of Mining and Technology, Xuzhou 221116, China; wangxinyu@cumt.edu.cn

**Keywords:** Smog Free Tower, air purification, housing price, moderating effect, traffic convenience

## Abstract

The Smog Free Tower (SFT) in the city of Xi’an, China, is the world’s first outdoor architecture that uses solar energy and filtration technology to purify polluted air. It provides a unique opportunity to explore residents’ willingness to pay for air quality and their related behaviors. Drawing on data collected after the establishment of the SFT, this paper reveals the characteristics of changes in people’s willingness to pay for clean air. We found that, prior to the release of an assessment report on the SFT, housing prices had an inverted U-shaped relationship with the distance to the SFT, which indicated people tended to purchase houses a certain distance away from the SFT. The threshold value of distance was inversely related to the greening ratio of the residential area. However, after the publication of the experimental report on the SFT, housing prices decreased as the distance to the SFT increased, indicating the closer the house was to the SFT, the more likely people were to buy it. These changes confirmed that people are willing to pay for clean air. The convenience of transportation had a significant moderating effect on the willingness to pay for clean air, however. In other words, people may buy houses with lower air quality if they have better transportation accessibility. The findings of this paper may have practical implications for environmental governance, urban planning, residential satisfaction, and real estate market regulation.

## 1. Introduction

Air pollution has increasingly become a worldwide public health concern [1,2]. It has been reported that air pollution not only increases the risk of various physical illnesses [2,3,4], such as respiratory diseases, neurodegenerative diseases, hypertension, cardiovascular diseases, and circulatory diseases, but can also induce severe insomnia and psychological problems [5,6]. Air pollution is more pronounced in metropolitan cities, where urban factors such as heavy automobile traffic and high population density produce more air pollution [6]. To prevent or minimize pollution damage, a series of measures have been taken against air pollution, including adding green spaces, more efficiently reducing emissions, the adjustment of the energy structure, and the development of alternative energy resources [7,8].

In China, air pollution has attracted public attention in recent decades, especially following the release of the documentary “Under the Dome” by Chai Jing in 2015 [9]. People’s awareness of air pollution has continued to increase as haze has become more frequent and serious in recent years. To protect themselves from hazardous haze, many citizens use air purifiers indoors and wear anti-smog face masks outdoors. The government has also enacted series of projects to build greener cities for citizens [10]. However, the governance of outdoor air pollution is more difficult than that of indoor pollution. Therefore, developing an outdoor technical system to absorb haze and purify the air has become an urgent and crucial issue.

To this end, the world’s first outdoor air purification building, which is known as the Smog Free Tower (SFT), was built in June 2016 in the city of Xi’an, China. The full name of the tower is the Solar-Assisted Large-Scale Cleaning System (SALSCS in short) [11,12]. It is the first architecture in the world to use solar energy and filtering techniques to clean polluted air [12]. Its basic operation principle is to inhale polluted air from the bottom of the tower first, heat the air by solar energy, then dispose of the air using a filter net and photocatalytic material, before lastly exporting fresh air through the diversion tower. The project group first unveiled an experimental report on the SFT in April 2018. It reported that the SFT was able to improve the air quality in a range of 10 km around the tower. The tower can reduce the concentration of PM_2.5_ particulate pollution by about 11 to 19 percent. Moreover, the neighborhoods closest to the SFT also benefit from the purification treatment, even if affected by polluted air inhaled by the tower clusters in areas near the SFT [13].

Air quality has increasingly become an important factor influencing citizens’ residential choices [14]. It has been shown that housing prices are higher in places with better air quality [15,16]. In other words, housing prices can be an important instrument to measure people’s willingness to pay for clean air, using the hedonic model [17,18]. As the first outdoor air-purifying tower, the SFT is a completely new concept, which could send divergent messages to residents at different stages. Therefore, the operation of the tower provides a unique opportunity to analyze the impacts of residents’ willingness to pay for clean air. However, despite much public attention on the SFT, there have been few published studies on the willingness to pay for clean air in this particular situation, except for one study based on data from January 2016 to June 2017, which revealed that the SFT had increased the housing prices of the purified area by 4% [19]. However, the cited study only focused on the completion period of the SFT. Thus, it may not have fully captured the impacts on housing prices caused by changes in public attitudes. Much more consideration should be given to the public’s responses in a longer observation window, especially after the release of the assessment report for the SFT. Did the publication of the test report increase people’s willingness to pay for clean air? Before the publication of the test report, to what extent did the risk of the uncertainty around the effectiveness and operation process of the SFT influence residents’ housing choices? Moreover, what role do traditional decisive variables, such as the greening ratio and transportation accessibility, play in the relationship between the distance to the SFT and housing prices? This study aimed to answer these questions and provide a renewed understanding of the willingness to pay for clean air based on data before and after the announcement of the assessment report of the SFT. Such research may help environment policymakers to consider the impacts of environmental improvement projects, and also enlighten people around practices related to real estate development, transportation, and urban planning in China and even the world.

In the following sections, the theoretical perspective and hypothesis development are illustrated. Based on the literature review, suitable variables, models, and data are outlined. Then, the housing prices of the affected area before and after the assessment report was released are presented. Further, hedonic models are employed to analyze how much people are willing to pay for clean air. Lastly, the main findings are summarized, and policy implications are also highlighted.

## 2. Review of the Literature and Hypothesis Development

Housing prices have long been a popular topic in urban studies. Unraveling the determinants of housing prices has attracted a significant amount of research attention. Transportation accessibility and neighborhood and structural characteristics are the key variables in determining housing prices [20,21,22,23]. In recent years, the positive effects of air quality on residents’ choice of residential location have drawn considerable attention [24]. Its attraction has grown with the rising concern about environment pollution. However, unlike the other three groups of variables, the measurement of residents’ willingness to pay for clean air is difficult, in that air quality has no fixed value as a type of public good [19]. Two major perspectives regarding the measurement of the economic value of clean air can be identified [19,25]. First, the stated preference approach posits that residents can accurately pinpoint their willingness to pay for different levels of air quality [26]. The contingent valuation model is generally employed to directly examine willingness to pay. However, the results obtained using this approach are likely to be biased because residents may not present their willingness correctly and objectively [19]. The revealed preference approach gauges the economic value of clean air through market data, mainly based on the hedonic price model. Existing research often used the impact on housing prices to evaluate willingness to pay [27,28]. This approach could also give biased results in view of the spatial self-selecting problem [19].

While a plethora of studies have delved into residents’ willingness to pay for clean air in developed countries, intellectual inquiries about the extent people are willing to pay for air quality in developing countries have just begun in recent years [19,29]. China has experienced unprecedented industrialization and urbanization in the past few decades, along with worsening air quality in most cities [30,31]. Scholars have reported on the spatial spillover effects of city-level air pollution on housing prices [32,33]. Nonetheless, there is a relatively small body of micro-data research concerning the willingness to pay for clean air. As an exception, Lan et al. (2020) argued that the extant studies suffer from self-selection bias, and suggested that the SFT provides a unique opportunity to address the self-selection problem [19]. Using the hedonic model, they calculated the net effect of the SFT on housing prices and revealed that the purification area’s housing prices have increased after the installment of the SFT. Based on the effectiveness of the SFT on changing housing prices in the above study, our present paper attempted to further explore the dynamics of residents’ willingness to pay for clean air and the moderating effects of three groups of variables on the relationship between air quality and housing prices. Specifically, four hypotheses concerning the relationship between the distance to the SFT and housing prices are proposed.

First, we proposed that the actual functioning of the SFT is critical to residents’ willingness to pay and related behavior. When the SFT was completed, the news media reported its general situation and function, which attracted wide public attention [34,35]. However, it can take time for people to trust new technology [36]. Before the assessment report of the tower’s trial operation was published, positive expectations of the effect of the SFT may not have formed due to residents’ concern regarding the potential negative impacts of the SFT. There was some concern that the polluted air absorbed by the SFT would aggregate in areas near the SFT, worsening the air pollution close to the SFT. The noise and radiation were also considered significant potential risks when choosing to live near the SFT. On the other hand, residents may have been interested in the opportunity to maximize access to clean air after the results of the trial operation were published. Thus, they may have tended to choose residential areas a certain distance away from the SFT, while areas within a closer area were of less interest. This means that there could have been a critical distance, rather than the closer the better being the rule, wherein if the distance to the SFT was less than the threshold, the housing prices could be expected to increase as the distance increased. Once beyond the threshold, the housing prices could be expected to decrease as the distance increased. Therefore, Hypothesis 1 of this study was proposed as follows:

**Hypothesis 1** **(H1).** *Before the release of the assessment report of the trial operation, a critical distance would have existed, and housing prices could be expected to rise and then fall with the distance.*

Although people tended to select houses located away from the critical distance, the value of the critical distance was also impacted by the greening ratio of the residential area. The important role of green plants in preventing and controlling air pollution has been well evidenced [37,38]. Green plants offer the absorption and purification of atmospheric pollutants in several ways, such as dust reduction, dust retention, dust absorption, dust fall, and dust prevention [38,39]. Thus, residential areas with plentiful greenery have a strong purifying ability of their own, which may have reduced concerns about the potential negative or limited effects of the SFT. On that account, living closer to the SFT may be more acceptable if the living area has a higher rate of greening. It seems the greening ratio acted as an insurance policy, ensuring maximum access to fresh air. Thus, based on Hypothesis 1, we proposed Hypothesis 2 as follows:

**Hypothesis 2** **(H2).** *Before the release of the assessment report of the trial operation, the value of the critical distance was inversely related to the greening ratio of the residential area.*

The assessment report was published at a press conference on 17 April 2018, by the Chinese Academy of Sciences, and was reported on in detail by the media. Most of the public’s questions were addressed at the press conference. The report demonstrated that the SFT effectively alleviated the haze by reducing 11% to 19% of the PM_2.5_ concentration level, and a surrounding area of 10 square kilometers benefited. Moreover, after the polluted air is sucked into the tower for purification, the clean air sinks and circulates from a height of 60 m, so the air is purified in the closest neighborhoods surrounding the SFT [11,12]. That is, polluted air does not accumulate in areas near the SFT. In addition, the report clarified that no radioactive materials were used during the construction and operation processes of the SFT, eliminating any potential radiation risks of living in the area close to the SFT. After the press conference, public uncertainty was reduced, and an understanding that the closer one lives to the SFT, the cleaner the air will be became widespread. Thus, the demand for houses close to the SFT was expected to increase due to the greater access to clean air. Accordingly, we proposed Hypothesis 3 as follows:

**Hypothesis 3** **(H3).** *After the release of the assessment report of the trial operation, housing prices decreased with increasing distance from the SFT.*

The release of the assessment report convinced the public that the closer they were to the SFT, the easier it would be to obtain clean air. However, since residential choice is influenced by many structural and environmental attributes, factors related to housing prices are complicated [22,40]. Locational convenience is one of the most important factors [40]. For example, people may prefer to live further away from the SFT for traffic convenience. Thus, people generally make a trade-off choice between living closer to the SFT and convenience [22]. Thus, it is reasonable to expect convenience to have a moderating role on the impact of clean air on housing prices. Hypothesis 4 in this study was as follows:

**Hypothesis 4** **(H4).** *After the release of the assessment report of the trial operation, the relationship between the distance to the SFT and housing prices was moderated by convenience.*

**Figure 1:**
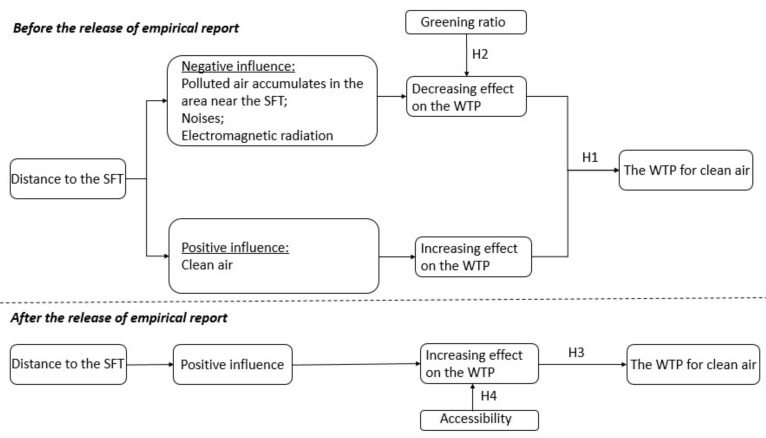
The proposed model (WTP: willingness to pay).

Figure 1 displays the conceptual model, which includes the four main testable hypotheses.

## 3. Methodology, Variables, and Data

To test the above four theoretical hypotheses, suitable methodologies, variables, and data were needed. In this section, we elaborate on the models used to capture the relationship between housing prices and the distance to the SFT, describe the relevant variables in detail, and show the characteristics of the selected data.

### 3.1. Model Specifications

As a universal model to capture housing buyers’ willingness to pay for various housing characteristics, the hedonic model was employed in this study. Following the literature, housing prices should be a function of a number of variables related to housing features and location [22,41,42].

The baseline hedonic model applied in this paper is given by:

(1) $$\begin{array}{cc} {\mathrm{HOUPRI}}_{\mathrm{it}}= & \alpha +\beta_{1}{\mathrm{DISTAN}}_{i}+\beta_{2}{\mathrm{DISTAN}}_{i}^{2}+\sum_{j=1}^{k}\gamma_{j}x_{ijt}+dummy_{circle}\\ & +dummy_{business}+dummy_{year}+dummy_{season}+\varepsilon_{it} \end{array}$$

In Equation (1), HOUPRI_it_ represents the housing price of neighborhood i at quarter t. DISTAN_i_ is the linear distance from neighborhood i to the SFT, and DISTAN_i_^2^ is the square of the distance. x_ijt_ is the value of the control variable j in neighborhood i at quarter t (the control variables include the number of households in the neighborhood, greening ratio, floor area ratio, number of bus stations, supermarkets, restaurants, banks, parks, schools, and hospitals within a 1 km radius). dummy_circle_ is the dummy variable for the loop line of the neighborhood, dummy_business_ is the dummy variable for the business district of the neighborhood, dummy_year_ is the dummy variable for the built year of the neighborhood, and dummy_season_ is the dummy variable for the season. α is the constant term, β_i_ and γ_j_ are the coefficients to be estimated, and ε_it_ is the error term. Notably, the logarithm of the housing price is applied in the regression models.

Despite the important effect of wind direction on the association between air quality and housing prices, the effect of wind direction could be ignored in the framework of this study for two reasons. First, unlike emissions that are clearly visible or have a pungent odor, clean air is difficult to detect by sight and smell [43]. In this context, residents tend to be more concerned about the distance to the SFT rather than the wind direction. Second, the prevailing wind direction in Xi’an is northeast and southwest, and the frequency of perennial static wind is 29% [44]. Thus, regardless of where a house is located around the SFT, it is difficult for people to balance the seasonal changes in wind direction. Wind direction was therefore not considered in our model specifications.

To capture the moderating effect of the greening ratio before the release of the assessment report, the interaction of distance and the greening ratio was added to Equation (1):

(2) $$\begin{array}{cc} HOUPRI_{it}= & \alpha +\beta_{1}(DISTAN_{i}\times GRERAT_{i})+\beta_{2}{\left(DISTAN_{i}\times GRERAT_{i}\right)}^{2}\\ & +\sum_{j=1}^{k}\gamma_{j}x_{ijt}+dummy_{circle}+dummy_{business}+dummy_{year}+dummy_{season}+\varepsilon_{it} \end{array}$$

where GRERAT_i_ is the greening ratio of neighborhood i. The meanings of the other symbols are the same as those in Equation (1).

To capture the moderating effects of convenience after the release of the assessment report, the interaction terms of the distance and convenience variables were added to the hedonic model:

(3) $$\begin{array}{cc} HOUPRI_{it}= & \alpha +\beta DISTAN_{i}+\sum_{j=1}^{k}\eta_{j}(DISTAN_{i}\times x_{ijt})+\sum_{j=1}^{k}\gamma_{j}x_{ijt}\\ & +dummy_{circle}+dummy_{business}+dummy_{year}+dummy_{season}+\varepsilon_{it} \end{array}$$

where DISTAN_i_ × x_ijt_ is the interaction term of the distance and convenience variables, and β and η_j_ are the coefficients to be estimated. The meanings of the other symbols are the same as those in Equation (1).

### 3.2. Variables and Data

**Figure 2:**
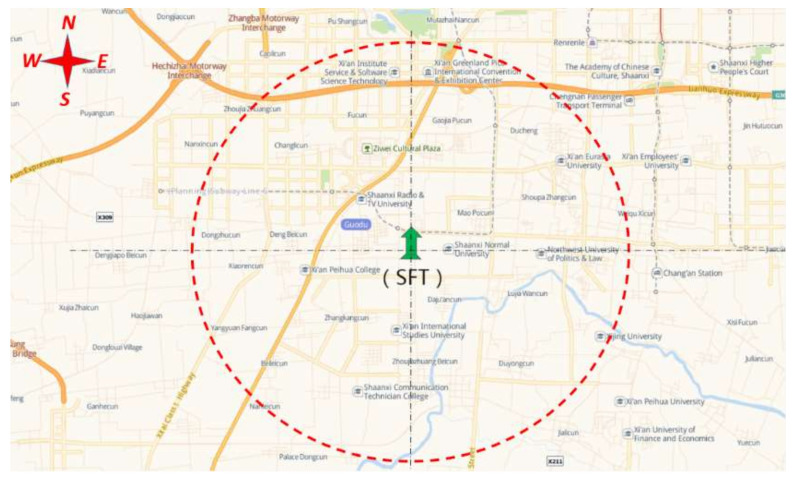
Research site.

**Figure 3:**
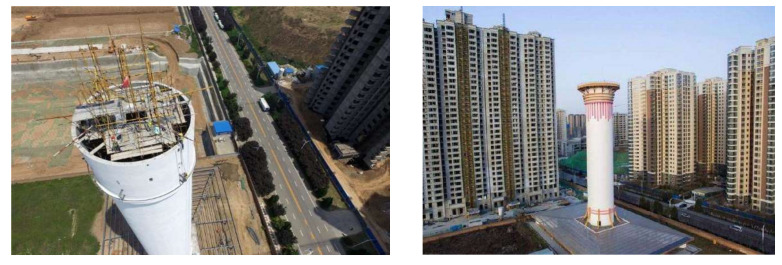
Pictures of the SFT (source: https://image.baidu.com, accessed on 12 July 2021).

The SFT was operated in August 2016, and the assessment report was released in April 2018. Therefore, the time frame of March 2017 to March 2018 was selected as the stage before the publication of the assessment report, and May 2018 to December 2018 as the stage after the publication of the assessment report. The research area in this paper included the neighborhoods inside a radius of about 5 km around the SFT, and the housing prices of these neighborhoods were observed during both stages. Figure 2 shows the geography of the research site. The SFT is located in Changan District, which is in the suburbs of the city of Xi’an. The appearance of the SFT and the surrounding environment is shown in Figure 3. This includes residential areas and varied types of neighborhoods, including newly built neighborhoods, old neighborhoods built before 2000, and neighborhoods under construction. After the demarcation of the boundary of the research area, 108 neighborhoods were selected. The data on second-hand housing prices and the control and dummy variables for the 108 neighborhoods were collected from the website Anjuke (https://xa.anjuke.com) (accessed on 20 January 2020), which is a large chain real estate company in China. The linear distance from the neighborhood to the SFT was gauged based on the Baidu electronic map. The definitions and statistics of the variables in this study are shown in Table 1.

## 4. Empirical Findings and Discussions

In the subsequent analysis, we first give detailed information on the dynamics of housing prices between March 2017 and December 2018. Then, the hedonic models specified above are applied to test the changes in the effects of distance to the SFT on housing prices before and after the release of the assessment report.

### 4.1. Descriptive Analysis

**Figure 4:**
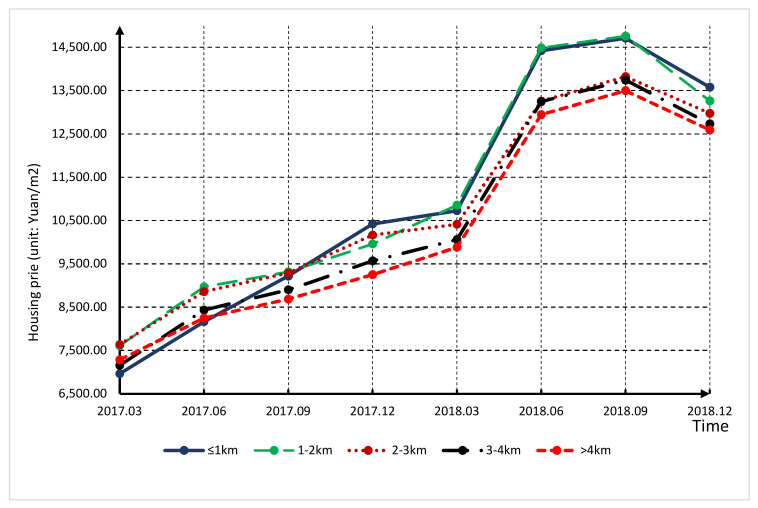
Average housing prices of different radiation areas from March 2017 to December 2018.

Based on the collected data, we calculated the average housing prices within 5 km of the SFT in each observational window. Figure 4 shows how the housing prices changed as the distance to the SFT increased from March 2017 to December 2018. Several observations can be derived from the figure. First, the prices of all neighborhoods show an obvious upward trend, which indicates the rising trend in the housing market in the city. Second, intuitively, housing prices inside the radius of 5 km have rapidly increased since the disclosure of the assessment report, especially in the area within a radius of 2 km. This validates our assumption that residents’ housing choice behavior would change due to the assessment report disclosure. Third, the housing prices of neighborhoods located less than 1 km from the SFT experienced complex dynamics; they were the lowest at the start of the observational window, but the highest at the end. This demonstrates how people’s willingness to pay for clean air increased with the operation process of the SFT.

### 4.2. Estimation Results and Discussions

Equations (1)–(3) were used to further test the hypotheses highlighted above. The estimation results of these models are presented in Table 2, Table 3, Table 4 and Table 5. In general, the results supported our hypotheses. As predicted, the change in people’s expectations regarding the effectiveness of the SFT leads to changes in their willingness to pay for clean air. After the confirmation of the effectiveness of the SFT, the distance to the SFT became a significant variable influencing housing prices. In addition, the ideal distance also depended on the greening ratio and transportation accessibility of the residential area.

#### 4.2.1. Association between the Distance to the SFT and Housing Prices before the Release of the Assessment Report

Table 2 shows the link between the distance to the SFT and housing prices. In Model 1 of Table 2, housing prices were only regressed on distance and the square of it. To obtain more accurate estimation results, we gradually added control variables in Models 2, 3, and 4. Further, we controlled for the general time trend effect by employing a time dummy for each season (dummy_season_) in Model 4. The four models showed that the square of the distance was significantly and negatively related to housing prices, which indicates that there is an inverted U-shaped relationship between the distance to the SFT and housing prices, and the relationship is robust. Model 4 shows the lowest value of the Root MSE but the highest R-squared, indicating the model with all dummies had the highest estimated accuracy. Then, the critical distance was calculated using the estimated coefficients of distance and the square in Model 4. It was revealed that the distance to the SFT was positively related to housing prices when the distance was shorter than 145 m, whereas it was negatively related to housing prices when the distance was greater than 145 m. This indicates that people tended to choose houses 145 m away from the tower and as close to it as possible, probably because people were worried about the poor purification effects when too close to the tower, but were not willing to lose the opportunity to obtain the maximum amount of clean air before the release of the assessment report. Therefore, Hypothesis 1 was confirmed.

Table 3 presents the moderating effect of the greening ratio on the relationship between the distance to the SFT and housing prices. Model 8 in Table 3 displays the lowest value of the Root MSE and the highest R-squared, indicating the model with all dummies had the highest estimated accuracy in Table 3. According to the coefficients in Model 8, we found that the critical distance is equal to 67.5/GRERAT, which revealed the critical distance was inversely related to the greening ratio. Taking the maximum value of the greening ratio in the sample, the critical distance was about 112.5 m. However, using the minimum value of the greening ratio in the sample, the critical distance was about 421.9 m. This means the ideal threshold of the distance ranges from about 113 to 422 m with changes to the greening ratio. Thus, the greening ratio indeed played a moderating role in the relationship between the distance to the SFT and housing prices. Hypothesis 2 was supported.

#### 4.2.2. Association between the Distance to the SWF and Housing Prices after the Release of the Assessment Report

Table 4 reports the relationship between distance and housing prices after the release of the trial result. It can be seen that the estimates of distance-squared are not significant, while the coefficients of distance are negatively and significantly related to housing prices except in Model 9 in Table 4. This indicates that people’s worries reduced and they thought the purifying tower was efficient after the release of the assessment report, which induced an increase in housing prices of neighborhoods located near the tower. Therefore, the closer to the SFT, the higher people’s willingness was to pay for clean air. Hypothesis 3 was confirmed.

Table 5 shows the moderating roles of various accessibilities in the relationship between distance and housing prices after the release of the trial result. Model 16 showed the lowest value of the Root MSE but the highest R-squared, which confirmed it had the highest estimation accuracy. It can be seen from Model 16 that the estimate of the distance was significantly negative, and the coefficient of the interaction of distance and the number of bus stops was significantly positive. Further, its value was much higher than the other interaction coefficients. This implies that, compared to air quality, residents are more sensitive to transportation accessibility. The number of bus stops is fewer in residential areas closer to the SFT. On average, the neighborhoods within 1 km from the tower have five bus stations; in comparison, neighborhoods 1 km away have about 6.3 bus stops. Thus, residents were more likely to choose houses with easily accessible transportation than clean air. In other words, people generally place more weight on transportation accessibility than on air quality. This suggests that the government should optimize the transportation conditions around the air purification tower to increase people’s residential satisfaction.

### 4.3. Implications of Results

Air quality has increasingly become an important factor influencing housing choices [18,19,24,45]. In confronting serious air pollution, it is common and efficient to control pollution from the source. While households can obtain clean air by installing indoor air purifiers, there is no mature technical method for the efficient purification of polluted air in outdoor public spaces at a large scale, in a recyclable and sustainable manner. Passive outdoor haze control technology systems do not target the source of pollution, and this needs to be considered in future research. However, the Smog Free Tower (SFT) in Xi’an is the first in the world, and so is considered a novel outdoor haze reduction experiment. Before the publication of the assessment report on the tower, people doubted the new technology. Our analysis evidenced that residents tended to buy houses a certain distance away from the SFT. This attitude changed, however, after the publication of the assessment report, which confirmed the effectiveness of the SFT and alleviated concerns about the potential risks.

The analysis of the responses of residents’ housing choice behavior to the SFT in the two stages revealed practical implications for building healthy cities, including for environmental governance, the real estate market, transportation, and urban planning. First, as haze is becoming increasingly serious, environmental protection measures are urgently needed to control it. The present paper confirmed people’s increased environmental awareness. It suggests that the public’s expectations of environmental governance are likely to change as the intermediate evaluation changes. Second, clean air has a positive capitalization effect on housing prices. In future, appropriate design environment policy, and the associated effects of such a policy on the housing market, should be considered. Third, this study highlighted the persistent importance of transportation accessibility and the greening ratio in housing choices. It suggested that the government should optimize the transportation conditions around air purification facilities to increase people’s satisfaction with living there.

## 5. Conclusions

This study attempted to reveal residents’ willingness to pay for clean air by using the unique quasi-natural experiment of the world’s first outdoor air purification building in Xi’an, China. This rare experiment not only overcomes self-selection bias, but also provides a valuable opportunity to distinguish dwellers’ behavior responses to air quality improvements at different stages. This study captured the changes in residents’ attitudes to the SFT, and the characteristics of their willingness to pay for clean air, through comparing the housing data before and after the publication of the assessment report for the tower. Simultaneously, the present study emphasized the moderating roles of the greening ratio and transportation accessibility in people’s pursuit of air quality. Hedonic models were employed to quantify the relative importance of the distance to the SFT and depict its changing relationship with housing prices. Specifically, the estimation results showed that before the publication of the assessment report, the distance to the SFT had an inverse U-shaped relationship with housing prices, and obvious threshold effects. Green plants can be regarded as the community’s own air purification facilities, as they have a protective effect against air pollution and can purify polluted air. The greening ratio of the residential area had a moderating effect on the non-linear relationship between the distance to the SFT and housing prices. After the publication of the assessment report, the distance to the SFT was negatively related to housing prices. The tendency of the housing price changes demonstrated that people are willing to pay for clean air. However, we found that transportation accessibility is more significant when selecting a residential location than clean air. That is, residents generally place more weight on transportation accessibility than on air quality when buying houses.

The present study contributes to the understanding of willingness to pay for clean air. We demonstrate how people’s expectations of the effectiveness of air purification change this willingness to pay. It is among the first to use a quasi-natural experiment to explain residents’ willingness to pay for air quality [19]. It uncovers the behavior dynamic in dwellers’ willingness to pay for clean air based on a longer observation window than in many existing studies. This particular experiment was able to overcome the traditional endogenous bias and provide more reliable analysis results. Furthermore, we find that improving transportation accessibility and the greening ratio increases the willingness to pay for clean air. This may help to improve theories on locational attainment. In addition, the moderating effects of transportation accessibility and greening ratio on the willingness to pay might have several implications for urban planners and policymakers.

Several limitations of this work should be acknowledged. First, we used the number of restaurants, supermarkets, banks, hospitals, and schools to measure the convenience of the neighborhood, but did not consider the quality of those surrounding services and facilities [40,41]. Second, important housing structure characteristics, such as the decoration degree and property management level, were not included in this study [40,46]. Third, as the present data were at the neighborhood level, we were not able to infer relationships between individual characteristics (e.g., income, education level, family structure) and willingness to pay for clean air. To overcome such limitations and provide a more complete picture of residents’ responses to the SFT and willingness to pay for clean air, we intend to complement the largely quantitative fieldwork by conducting a large-scale survey and qualitative in-depth interviews in the future.

## Figures and Tables

**Table 1:** Definition and descriptive statistics of the variables.

Variable	Definition	Unit or Coding	Mean	St. Dev.	Minimum	Maximum
HOUPRI	Average unit price of the neighborhood	Yuan/m^2^	10,353.53	4625.321	1800	32,673
DISTAN	Distance to the SWF	km	3.33	1.325	0.37	5.1
HOUHOL	Neighborhood size	households	1537.99	2038.521	36	12,746
GRERAT	Greening ratio	%	36.64	8.180	16	60
FAR	Floor area ratio	%	3.42	1.309	0.96	10.3
BUSTOP	Number of bus stops	PCs	6.20	1.977	1	13
SUPMAR	Number of supermarkets	PCs	7.21	2.869	1	14
RESTAU	Number of restaurants	PCs	6.67	4.481	0	16
BANK	Number of banks	PCs	6.01	3.929	0	24
PARK	Number of parks	PCs	1.06	1.030	0	4
SCHOOL	Number of schools	PCs	5.21	2.949	0	14
HOSPIT	Number of hospitals	PCs	2.24	2.565	0	11
SECRIN	Whether located within the second ring (Yes = 1, No = 0)	—	0.01	0.096	0	1
SECTHI	Whether located between the second and third ring (Yes = 1, No = 0)	—	0.21	0.410	0	1
BEYTHI	Whether located outside of the third ring (Yes = 1, No = 0)	—	0.77	0.422	0	1

Note: — indicates that the corresponding variable is unitless. Due to limited space, season dummy variables (8), business district dummy variables (19), and built year dummy variables (23) are not included in the table. They can be found in the Appendix A Table A1. The included bus stops, supermarkets, restaurants, banks, parks, schools, and hospitals are all within 1 km of the neighborhoods.

**Table 2:** Link between the distance to the SFT and housing prices before the release of the assessment report.

	Model 1	Model 2	Model 3	Model 4
	HOUPRI	HOUPRI	HOUPRI	HOUPRI
DISTAN	0.09146	0.05124	0.04663	0.06672
	(0.07267)	(0.05297)	(0.05982)	(0.05247)
DISTAN^2^	−0.14607 ***	−0.19027 **	−0.19397 **	−0.23079 ***
	(0.04452)	(0.08232)	(0.09080)	(0.07987)
HOUHOL		0.08676 ***	0.07006 ***	0.07089 ***
		(0.01225)	(0.01315)	(0.01161)
GRERAT		0.00030	0.00137	0.00238
		(0.00195)	(0.00241)	(0.00215)
FAR		−0.04047 ***	−0.05460 ***	−0.05304 ***
		(0.01059)	(0.01225)	(0.01072)
BUSTOP		0.10697 **	0.12980 ***	0.10775 ***
		(0.04422)	(0.04396)	(0.03915)
SUPMAR		−0.01296 **	−0.00923 *	−0.00985 **
		(0.00521)	(0.00559)	(0.00491)
RESTAU		−0.00082	0.00610	0.00581
		(0.00431)	(0.00479)	(0.00419)
BANK		0.00958 **	0.01448 ***	0.01182 **
		(0.00444)	(0.00517)	(0.00457)
PARK		0.01388	0.03416 **	0.02093
		(0.01518)	(0.01689)	(0.01526)
SCHOOL		−0.01257 **	0.00181	0.00097
		(0.00601)	(0.00678)	(0.00592)
HOSPIT		0.00046	0.01072	0.02143 **
		(0.00921)	(0.00958)	(0.00878)
dummy_business_		Yes	Yes	Yes
dummy_year_			Yes	Yes
dummy_season_				Yes
dummy_circle_				Yes
C	9.10578 ***	8.55806 ***	8.06303 ***	7.73887 ***
	(0.03889)	(0.14620)	(0.19919)	(0.22661)
N	540	540	540	540
F	12.90515	23.53453	18.48574	24.04787
Root MSE	0.39413	0.27077	0.24719	0.21589
R-squared	0.04586	0.57233	0.65893	0.74357

Note: *, **, and *** indicate statistical significance at the 10%, 5%, and 1% levels, respectively. Standard errors are in parentheses.

**Table 3:** The moderating role of the greening ratio before the release of the assessment report.

	Model 5	Model 6	Model 7	Model 8
	HOUPRI	HOUPRI	HOUPRI	HOUPRI
DISTAN × GRERAT	0.00566 ***	0.00221 *	0.00328 **	0.00299 ***
	(0.00080)	(0.00124)	(0.00131)	(0.00115)
(DISTAN × GRERAT)^2^	−0.02955 ***	−0.01698 ***	−0.02256 ***	−0.02216 ***
	(0.00385)	(0.00577)	(0.00607)	(0.00529)
Control Variables		Yes	Yes	Yes
dummy_business_		Yes	Yes	Yes
dummy_year_			Yes	Yes
dummy_season_				Yes
dummy_circle_				Yes
C	8.86052 ***	8.59337 ***	8.38673 ***	8.02393 ***
	(0.04361)	(0.36108)	(0.44121)	(0.39258)
N	540	540	540	540
F	29.41852	23.03047	18.59793	24.76161
Root MSE	0.38305	0.26993	0.24520	0.21355
R-squared	0.09875	0.57580	0.66508	0.74911

Note: *, **, and *** indicate statistical significance at the 10%, 5%, and 1% levels, respectively. Standard errors are in parentheses. The control variables are the same as in Table 2.

**Table 4:** The relationship between the distance and housing prices after the release of the assessment report.

	Model 9	Model 10	Model 11	Model 12
	HOUPRI	HOUPRI	HOUPRI	HOUPRI
DISTAN	−0.03916	−0.13825 **	−0.15634 **	−0.12153 *
	(0.08206)	(0.06156)	(0.06889)	(0.06994)
DISTAN^2^	−0.00513	0.00113	0.00035	−0.00123
	(0.00534)	(0.00456)	(0.00477)	(0.00484)
Control Variables		Yes	Yes	Yes
dummy_business_		Yes	Yes	Yes
dummy_year_			Yes	Yes
dummy_season_				Yes
dummy_circle_				Yes
C	9.52623 ***	8.56181 ***	8.03078 ***	8.01684 ***
	(0.04537)	(0.17654)	(0.25695)	(0.25425)
N	324	324	324	324
F	5.07284	20.10844	14.87738	14.36817
Root MSE	0.34820	0.21165	0.19605	0.19356
R-squared	0.03064	0.67308	0.74058	0.75084

Note: *, **, and *** indicate statistical significance at the 10%, 5%, and 1% levels, respectively. Standard errors are in parentheses. The control variables are the same as in Table 2.

**Table 5:** The moderating roles of accessibilities after the release of the assessment report.

	Model 13	Model 14	Model 15	Model 16
	HOUPRI	HOUPRI	HOUPRI	HOUPRI
DISTAN	−0.19616	−0.40333 *	−0.44946 *	−0.62495 **
	(0.20141)	(0.23198)	(0.24382)	(0.25382)
DISTAN × BUSTOP	0.06071	0.15067 *	0.17054 *	0.20703 **
	(0.07273)	(0.08189)	(0.08824)	(0.09046)
DISTAN × SUPMAR	0.06140	−0.04347	−0.05022	0.01202
	(0.07566)	(0.10260)	(0.10270)	(0.10528)
DISTAN × RESTAU	−0.01030	−0.00983	−0.01281	−0.01796 *
	(0.00745)	(0.00802)	(0.00945)	(0.00966)
DISTAN × BANK	0.03497 ***	0.03732 ***	0.03732 ***	0.03154 **
	(0.01145)	(0.01425)	(0.01418)	(0.01428)
DISTAN × PARK	−0.01574	0.03306	0.02737	0.02981
	(0.03286)	(0.03961)	(0.04058)	(0.04123)
DISTAN × SCHOOL	−0.03510 ***	−0.00842	−0.00324	0.00033
	(0.01153)	(0.01516)	(0.01748)	(0.01741)
DISTAN × HOSPIT	−0.02443	−0.02850	−0.02503	−0.00511
	(0.02905)	(0.03624)	(0.03652)	(0.03717)
Control Variables	Yes	Yes	Yes	Yes
dummy_business_	Yes	Yes	Yes	Yes
dummy_year_		Yes	Yes	Yes
dummy_season_			Yes	Yes
dummy_circle_				Yes
C	8.27132 ***	7.98072 ***	8.08150 ***	8.28313 ***
	(0.35942)	(0.40710)	(0.44657)	(0.45350)
N	324	324	324	324
F	18.28400	14.14256	13.67857	13.53692
Root MSE	0.20610	0.19352	0.19248	0.19102
R-squared	0.69637	0.75189	0.75732	0.76279

Note: *, **, and *** indicate statistical significance at the 10%, 5%, and 1% levels, respectively. Standard errors are in parentheses. The control variables are the same as in Table 2.

## Data Availability

Data that support the findings of this study are available from the corresponding author upon reasonable request.

## Appendix A

**Table A1:** Definition and descriptive statistics of the dummy variables not included in Table 1.

Variable	Definition	Unit or Coding	Mean	St. Dev.	Minimum	Maximum
S20173	Whether it is the first quarter of 2017. (Yes = 1, No = 0)	—	0.13	0.331	0	1
S20176	Whether it is the second quarter of 2017. (Yes = 1, No = 0)	—	0.13	0.331	0	1
S20179	Whether it is the third quarter of 2017. (Yes = 1, No = 0)	—	0.13	0.331	0	1
S201712	Whether it is the fourth quarter of 2017. (Yes = 1, No = 0)	—	0.13	0.331	0	1
S20183	Whether it is the first quarter of 2018. (Yes = 1, No = 0)	—	0.13	0.331	0	1
S20186	Whether it is the second quarter of 2018. (Yes = 1, No = 0)	—	0.13	0.331	0	1
S20189	Whether it is the third quarter of 2018. (Yes = 1, No = 0)	—	0.13	0.331	0	1
S201812	Whether it is the fourth quarter of 2018. (Yes = 1, No = 0)	—	0.13	0.331	0	1
XIZHAI	Whether located in the business district Xizhai (Yes = 1, No = 0)	—	0.06	0.229	0	1
GUODU	Whether located in the business district Guodu (Yes = 1, No = 0)	—	0.22	0.416	0	1
XCAJ	Whether located in the business district Chang’an Street (Yes = 1, No = 0)	—	0.08	0.277	0	1
ZIWU	Whether located in the business district Ziwu (Yes = 1, No = 0)	—	0.01	0.096	0	1
XIFENG	Whether located in the business district Xifeng (Yes = 1, No = 0)	—	0.05	0.210	0	1
DAXUCH	Whether located in the business district Daxue (Yes = 1, No = 0)	—	0.17	0.373	0	1
DIZICH	Whether located in the business district Dianzi (Yes = 1, No = 0)	—	0.05	0.210	0	1
ZWTYDS	Whether located in the business district Ziwei (Yes = 1, No = 0)	—	0.03	0.164	0	1
WEIQU	Whether located in the business district Weiqu (Yes = 1, No = 0)	—	0.17	0.373	0	1
KEJI	Whether located in the business district Keji (Yes = 1, No = 0)	—	0.03	0.164	0	1
JINYE	Whether located in the business district Jinye (Yes = 1, No = 0)	—	0.06	0.229	0	1
CHANSQ	Whether located in the business district Chang’an Square (Yes = 1, No = 0)	—	0.01	0.096	0	1
ZHBAXI	Whether located in the business district Zhangba (Yes = 1, No = 0)	—	0.02	0.135	0	1
DZJIE	Whether located in the business district Dianzi Street (Yes = 1, No = 0)	—	0.01	0.096	0	1
LIMEXICH	Whether located in the business district Lianmeng (Yes = 1, No = 0)	—	0.01	0.096	0	1
XIMEYU	Whether located in the business district Rongchuang (Yes = 1, No = 0)	—	0.01	0.096	0	1
YAHUZH	Whether located in the business district Yanhuan (Yes = 1, No = 0)	—	0.01	0.096	0	1
MINGDE	Whether located in the business district Mingde (Yes = 1, No = 0)	—	0.01	0.096	0	1
GXYIZH	Whether located in the business district Gaoxin (Yes = 1, No = 0)	—	0.01	0.096	0	1
Y1999	Whether the neighborhood was completed in 1999. (Yes = 1, No = 0)	—	0.01	0.096	0	1
Y2000	Whether the neighborhood was completed in 2000. (Yes = 1, No = 0)	—	0.01	0.096	0	1
Y2001	Whether the neighborhood was completed in 2001. (Yes = 1, No = 0)	—	0.02	0.135	0	1
Y2002	Whether the neighborhood was completed in 2002. (Yes = 1, No = 0)	—	0.01	0.096	0	1
Y2003	Whether the neighborhood was completed in 2003. (Yes = 1, No = 0)	—	0.03	0.164	0	1
Y2004	Whether the neighborhood was completed in 2004. (Yes = 1, No = 0)	—	0.04	0.189	0	1
Y2005	Whether the neighborhood was completed in 2005. (Yes = 1, No = 0)	—	0.02	0.135	0	1
Y2006	Whether the neighborhood was completed in 2006. (Yes = 1, No = 0)	—	0.04	0.189	0	1
Y2007	Whether the neighborhood was completed in 2007. (Yes = 1, No = 0)	—	0.05	0.210	0	1
Y2008	Whether the neighborhood was completed in 2008. (Yes = 1, No = 0)	—	0.06	0.246	0	1
Y2009	Whether the neighborhood was completed in 2009. (Yes = 1, No = 0)	—	0.06	0.246	0	1
Y2010	Whether the neighborhood was completed in 2010. (Yes = 1, No = 0)	—	0.10	0.303	0	1
Y2011	Whether the neighborhood was completed in 2011. (Yes = 1, No = 0)	—	0.06	0.246	0	1
Y2012	Whether the neighborhood was completed in 2012. (Yes = 1, No = 0)	—	0.06	0.246	0	1
Y2013	Whether the neighborhood was completed in 2013. (Yes = 1, No = 0)	—	0.05	0.210	0	1
Y2014	Whether the neighborhood was completed in 2014. (Yes = 1, No = 0)	—	0.09	0.290	0	1
Y2015	Whether the neighborhood was completed in 2015. (Yes = 1, No = 0)	—	0.10	0.303	0	1
Y2016	Whether the neighborhood was completed in 2016. (Yes = 1, No = 0)	—	0.02	0.135	0	1
Y2017	Whether the neighborhood was completed in 2017. (Yes = 1, No = 0)	—	0.07	0.262	0	1
Y2018	Whether the neighborhood was completed in 2018. (Yes = 1, No = 0)	—	0.03	0.164	0	1
Y2019	Whether the neighborhood was completed in 2019. (Yes = 1, No = 0)	—	0.03	0.164	0	1
Y2020	Whether the neighborhood was completed in 2020. (Yes = 1, No = 0)	—	0.03	0.164	0	1
Y2021	Whether the neighborhood was completed in 2021. (Yes = 1, No = 0)	—	0.01	0.096	0	1

Note: — indicates that the corresponding variable is unitless.

## References

[1] Pope C.A., Douglas W.D. (2006). Health Effects of Fine Particulate Air Pollution: Lines That Connect. J. Air Waste Manag. Assoc..

[2] Pope C.A., Burnett R.T., Thun M.J., Calle E.E., Krewski D., Ito K., Thurston G.D. (2002). Lung Cancer, Cardiopulmonary Mortality, and Long-Term Exposure to Fine Particulate Air Pollution. JAMA.

[3] Gautam D., Bolia N.B. (2020). Air pollution: Impact and interventions. Air Qual. Atmos. Health.

[4] Brunekreef B., Holgate S.T. (2002). Air Pollution and Health. Lancet.

[5] Heyes A., Zhu M. (2019). Air pollution as a cause of sleeplessness: Social media evidence from a panel of Chinese cities. J. Environ. Econ. Manag..

[6] Wang R., Xue D., Liu Y., Liu P., Chen H. (2018). The Relationship between Air Pollution and Depression in China: Is Neighbourhood Social Capital Protective?. Int. J. Environ. Res. Public Health.

[7] Yang X., Zhang J., Ren S., Ran Q. (2020). Can the new energy demonstration city policy reduce environmental pollution? Evidence from a quasi-natural experiment in China. J. Clean. Prod..

[8] Xie Y., Wu D., Zhu S. (2020). Can new energy vehicles subsidy curb the urban air pollution? Empirical evidence from pilot cities in China. Sci. Total. Environ..

[9] Tan Z. (2017). Smog Free Tower: Studio Roosegaarde, Beijing, September 2016–Present. Technol. Archit. Des..

[10] Zhou Q., Zhang X., Shao Q., Wang X. (2019). The non-linear effect of environmental regulation on haze pollution: Empirical evidence for 277 Chinese cities during 2002–2010. J. Environ. Manag..

[11] Cao Q., Huang M., Kuehn T.H., Shen L., Tao W.-Q., Cao J., Pui D.Y. (2018). Urban-scale SALSCS, Part II: A Parametric Study of System Performance. Aerosol Air Qual. Res..

[12] Cao Q., Kuehn T.H., Shen L., Chen S.-C., Zhang N., Huang Y., Cao J., Pui D.Y. (2018). Urban-scale SALSCS, Part I: Experimental Evaluation and Numerical Modeling of a Demonstration Unit. Aerosol Air Qual. Res..

[13] The Only Smog Free Tower in the world. http://news.hsw.cn/system/2018/0418/979401.shtml?rand=r8Lx5fEb.

[14] Chay K.Y., Greenstone M. (2005). Does air quality matter? Evidence from the housing market. J. Polit. Econ..

[15] Smith V.K., Huang J.C. (1993). Hedonic models and air pollution: Twenty-five years and counting. Environ. Resour. Econ..

[16] Zabel J.E., Kiel K.A. (2000). Estimating the Demand for Air Quality in Four U.S. Cities. Land Econ..

[17] Harrison D., Rubinfeld D.L. (1978). Hedonic housing prices and the demand for clean air. J. Environ. Econ. Manag..

[18] Won K.C., Phipps T.T., Anselin L. (2003). Measuring the benefits of air quality improvement: A spatial hedonic approach. J. Environ. Econ. Manag..

[19] Lan F., Lv J., Chen J., Zhang X., Zhao Z., Pui D.Y. (2020). Willingness to pay for staying away from haze: Evidence from a quasi-natural experiment in Xi’an. J. Environ. Manag..

[20] Can A. (1992). Specification and estimation of hedonic housing price models. Reg. Sci. Urban Econ..

[21] Efthymiou D., Antoniou C. (2013). How do transport infrastructure and policies affect house prices and rents? Evidence from Athens, Greece. Transp. Res. Part A Policy Pract..

[22] Li H., Wei Y.D., Yu Z., Tian G. (2016). Amenity, accessibility and housing values in metropolitan USA: A study of Salt Lake County, Utah. Cities.

[23] Smersh G.T., Smith M.T. (2000). Accessibility Changes and Urban House Price Appreciation: A Constrained Optimization Approach to Determining Distance Effects. J. Hous. Econ..

[24] Jim C., Chen W. (2007). Consumption preferences and environmental externalities: A hedonic analysis of the housing market in Guangzhou. Geoforum..

[25] Adamowicz W., Louviere J., Williams M. (1994). Combining Revealed and Stated Preference Methods for Valuing Environmental Amenities. J. Environ. Econ. Manag..

[26] Luechinger S. (2009). Valuing Air Quality Using the Life Satisfaction Approach. Econ. J..

[27] Cobb S.A. (1977). Site rent, air quality, and the demand for amenities. J. Environ. Econ. Manag..

[28] Zheng S., Kahn M., Liu H. (2010). Towards a System of open cities in China: Home prices, FDI flows and air quality in 35 major cities. Reg. Sci. Urban Econ..

[29] Zheng S., Cao J., Kahn M.E., Sun C. (2014). Real estate valuation and cross-boundary air pollution externalities: Evidence from Chinese cities. J. Real Estate Financ. Econ..

[30] Rohde R.A., Muller R.A. (2015). Air Pollution in China: Mapping of Concentrations and Sources. PLoS ONE.

[31] Xie Y., Dai H., Dong H., Hanaoka T., Masui T. (2016). Economic Impacts from PM 2.5 Pollution-Related Health Effects in China: A Provincial-Level Analysis. Environ. Sci. Technol..

[32] Zhang H., Chen J., Wang Z. (2021). Spatial Heterogeneity in Spillover Effect of Air Pollution on Housing Prices: Evidence from China. Cities.

[33] Chen S., Jin H. (2018). Pricing for the clean air: Evidence from Chinese housing market. J. Clean. Prod..

[34] The Smog Free Tower in the City of Xi’an. http://news.cctv.com/2016/05/17/VIDEM1LWsSImujfeU24PHqUM160517.shtml.

[35] The Smog Free Tower in Xi’an Is Rooted. https://www.sohu.com/a/75289553_119038..

[36] Zhou H. (2014). Difficulties to overcome the adoration of GDP—four conceptual disorder during the development and their errorness nature. People’s Trib..

[37] Morancho A.B. (2003). A hedonic valuation of urban green areas. Landsc. Urban Plan..

[38] Anderson L.M., Cordell H.K. (1988). Influence of Trees on Residential Property Values in Athens, Georgia (USA): A Survey Based on Actual Sales Prices. Landsc. Urban Plan..

[39] Gromke C., Ruck B. (2007). Influence of Trees on the Dispersion of Pollutants in an Urban Street Canyon–Experimental Investigation of the Flow and Concentration Field. Atmos. Environ..

[40] Duan J., Tian G., Yang L., Zhou T. (2021). Addressing the Macroeconomic and Hedonic Determinants of Housing Prices in Beijing Metropolitan Area, China. Habitat Int..

[41] Geng B., Bao H., Liang Y. (2015). A study of the effect of a high-speed rail station on spatial variations in housing price based on the hedonic model. Habitat Int..

[42] Sirmans S., Macpherson D., Zietz E. (2005). The Composition of Hedonic Pricing Models. J. Real Estate Lit..

[43] Anstine J. (2003). Property Values in a Low Populated Area when Dual Noxious Facilities are Present. Growth Chang..

[44] Zhao J., Wang Q. (2010). The impact of wind projecting angles on energy consumption of buildings—The case from Xi’an. Urban Probl..

[45] Chen J., Hao Q., Yoon C. (2018). Measuring the welfare cost of air pollution in Shanghai: Evidence from the housing market. J. Environ. Plan. Manag..

[46] Wen H., Zhang Y., Zhang L. (2015). Assessing amenity effects of urban landscapes on housing price in Hangzhou, China. Urban For. Urban Green..

